# On Euthanasia

**Published:** 1916-07-01

**Authors:** 


### ON EUTHANASIA.

It is a commonplace to say that an important function of the medical practitioner is to relieve pain and suffering. Yet occasions arise in which the discharge of this duty raises some ethical difficulty. The question appears when a patient is known to be, or is believed to be, the victim of an incurable illness which is attended with pain or other form of bodily distress. On the one hand are those who feel that almost at any price relief must be obtained, and on the other is the view that to cloud the mind by powerful soporific drugs is in the circumstances a serious responsibility to accept. The issue is presented rather in acute cases than in those where an illness runs a prolonged and chronic course. In this latter event the element of time weighs largely, for it is possible to lessen the total suffering and yet to leave opportunity for the patient to exercise his mental activities in an approximately normal fashion. Hence there is little difficulty, and the practitioner will hardly shrink from steps which secure some measure of relief both for the patient and his friends. But when circumstances are more urgent decision is not always easy, and it is perhaps impossible to state an absolute rule. There may be in one case a degree of suffering which outweighs all other considerations, and in another there may be claims which must receive attention even at the cost of distress to all concerned.

One principle which commands general if not universal assent is that the practitioner may not deliberately take any step which is likely to shorten the patient's life. The duty of the profession is to prolong life, not to shorten it, and any departure from such a position is full of danger. There are at times temptations which humanity appears to prompt. But they are always to be resisted. The commanding fact of the situation is that at the best the verdict of the practitioner is a fallible one. However hopeless the outlook may seem there is the possibility of an error of judgment. The most experienced have before now made a mistake in prognosis, and in view of this an irrevocable decision cannot be defended. Again, once leave the sure ground of duty defined as an attempt to prolong life, and no principle of action can be found. The practitioner would be destitute of all rational guidance and would find himself tossed on a sea of uncertainty and doubt. Finally it may be remembered that symptoms which are very distressing to an onlooker may, and often do, mean no suffering to the patient. The laboured and noisy breathing which so often accompanies a deathbed scene is at times a source of great pain to the relatives, but it is practically certain that the dying man does not share this pain. It is a fair thing to urge this statement with great confidence and so to relieve the anguish of those who are torn by the contemplation of what seems to them acute distress. In any event there can be no acceptance by a medical practitioner of a responsibility which would anticipate the decrees of fate.

